# Changes in plasma inflammatory injury factors and their clinical significance in pediatric bacterial pneumonia and sepsis

**DOI:** 10.1515/biol-2025-1344

**Published:** 2026-07-31

**Authors:** Xia An, Haihua Chen, Zijun Ye

**Affiliations:** Department of Pediatrics, Huai’an Maternal and Child Health Care Hospital Affiliated to Yangzhou University, Huai’an 223002, Jiangsu Province, China

**Keywords:** bacterial pneumonia, sepsis, PCT, CRP, neutrophils, inflammatory injury factors

## Abstract

Bacterial pneumonia and sepsis are common severe infectious diseases in pediatrics. The overlapping early clinical manifestations of these two conditions often lead to misdiagnosis. Establishing a precise early differential diagnostic method is crucial for improving patient outcomes. This retrospective cohort study enrolled 126 children with bacterial pneumonia, 116 children with sepsis, and 65 healthy children as controls. Plasma procalcitonin (PCT), C-reactive protein (CRP), and peripheral blood leukocyte subset indicators were measured, and their differential diagnostic efficacy was analyzed. The results showed that PCT, CRP, white blood cell count, and absolute neutrophil count (NE#) were significantly higher in the sepsis group than in the bacterial pneumonia group (*P* < 0.05). The area under the curve for PCT in differentiating sepsis was 0.85, with an optimal cut-off value of 5.8 ng/mL. The optimal cut-off values for CRP in diagnosing sepsis and bacterial pneumonia were 28.5 mg/L and 20.0 mg/L, respectively, which could help distinguish local from systemic infection. The diagnostic value of NE# was significantly superior to that of the neutrophil percentage (AUC: 0.79 vs. 0.65). The sepsis group also had a longer disease course and a higher proportion of patients with a history of previous hospitalization (*P* < 0.05). The study confirmed that PCT and CRP have significant differential diagnostic value for pediatric bacterial pneumonia and sepsis, with PCT showing superior efficacy for identifying sepsis. The differentiated CRP cut-off values could reflect infection severity, and NE# was more suitable as an inflammatory assessment indicator in pediatric infections. Combining these biomarkers with clinical features like disease course and prior hospitalization history can guide early and precise clinical differentiation, providing a reference for timely targeted anti-infective therapy.

## 1 Introduction

Bacterial pneumonia and sepsis are common severe infectious diseases threatening children’s health. Young children are particularly vulnerable to both conditions due to their underdeveloped immune systems and unique respiratory anatomy [1], 2]. Sepsis often occurs secondary to underlying diseases or immunocompromised states, progresses rapidly, and poses significant diagnostic and therapeutic challenges. Bacterial pneumonia is characterized by local pulmonary inflammation. The early clinical manifestations of these two entities overlap considerably, frequently leading to clinical misdiagnosis [3]. Given the escalating challenge of antimicrobial resistance and the increasing difficulties associated with empirical treatment, the failure to promptly and accurately differentiate between these two conditions can delay targeted therapy, exacerbate the illness, and even become life-threatening [4], 5].

In clinical practice, procalcitonin (PCT), C-reactive protein (CRP), and white blood cell (WBC) subsets are commonly used inflammatory markers for evaluating infectious diseases. However, the specific value of these markers in differentiating pediatric bacterial pneumonia from sepsis lacks systematic clinical validation [6]. Existing studies have not definitively compared the diagnostic efficacy of absolute neutrophil count (NE#) versus neutrophil percentage (NE%), and standardized, differentiated diagnostic thresholds for the pediatric population have not been established, failing to meet the demands of precision medicine [7], [8], [9]. Furthermore, the diagnosis of pediatric sepsis often faces challenges related to the applicability of assessment tools derived from adults. The classic SOFA score was not designed for the pediatric population and lacks adaptation to children’s physiological characteristics. The clinical application of the pediatric-specific sequential organ failure assessment (pSOFA) score still requires supporting inflammatory biomarkers for diagnosis, and this gap contributes to persistent clinical uncertainty in the early differentiation between pediatric sepsis and bacterial pneumonia [10].

This study aimed to compare plasma PCT, CRP levels, and leukocyte subset indicators among children with bacterial pneumonia, children with sepsis, and healthy children. It sought to clarify the differential diagnostic value and optimal cut-off values of these indicators. By addressing the specific needs of pediatric diagnostic assessment, this research provides evidence-based support for the early and precise differentiation between pediatric bacterial pneumonia and sepsis, thereby optimizing clinical decision-making.

## 2 Materials and methods

### 2.1 Subjects

A total of 126 children with bacterial pneumonia and 116 children with sepsis were retrospectively included from the Pediatric ICU of Huai’an Maternal and Child Health Care Hospital Affiliated to Yangzhou University from January 2022 to December 2023. Additionally, 65 healthy children who underwent physical examinations in the hospital during the same period were enrolled as the control group (CG).

Case confirmation criteria: bacterial pneumonia group (BPG): priority was given to enrolling pathogen-positive cases (isolation of pathogens such as *Streptococcus pneumoniae*, *Haemophilus influenzae*, or *Staphylococcus aureus* from sputum culture or bronchoalveolar lavage fluid culture). For culture-negative but clinically confirmed cases, inclusion required meeting all of the following three criteria: i) clinical manifestations consistent with bacterial pneumonia (persistent high fever ≥38.5 °C, cough with purulent sputum, and auscultatory wet rales in the lungs); ii) chest imaging (CT/chest X-ray) showing definite pulmonary infiltrates; iii) PCT >0.5 ng/mL and significant symptomatic improvement after 48–72 h of antibiotic therapy, with concurrent exclusion of non-bacterial infections such as viral or mycoplasma infections (confirmed by negative respiratory virus antigen tests and negative *Mycoplasma* IgM antibody tests). A total of 21 culture-negative cases meeting these criteria were included, accounting for 16.7 % of the total BPG cases. Sepsis Group: All cases met the dual criteria of positive etiology or a confirmed infection focus plus organ dysfunction. Confirmed etiological cases required a positive culture from blood or an infection focus (e.g., pleural fluid, cerebrospinal fluid). Clinically highly suspected but etiologically unconfirmed cases had to simultaneously meet the following criteria: ① Presence of a definite infection focus (pneumonia, urinary tract infection, etc.); ② Pediatric-specific sequential organ failure assessment (pSOFA) score ≥ 2 (indicating organ dysfunction, e.g., respiratory rate > 30/min, systolic blood pressure < 90 mmHg, serum creatinine > 70 μmol/L); ③ Significantly elevated inflammatory markers (PCT > 2 ng/mL, CRP > 20 mg/L). A total of 18 clinically confirmed sepsis cases meeting these criteria were included, accounting for 15.5 % of the total SG cases. All cases were independently reviewed and confirmed by two senior pediatric attending physicians (each with ≥5 years of experience in infectious diseases). Discrepancies in assessment were resolved through departmental case discussions to minimize diagnostic bias.

Organ dysfunction (*e.g.,* respiratory failure: oxygenation index < 300 mmHg; circulatory impairment: capillary refill time > 2 s or hypotension; neurological impairment: altered mental status); (4) CG inclusion required meeting all of the following criteria: i) no infection-related symptoms (*e.g.,* fever, cough, diarrhea) within the past 3 months; ii) normal physical examination findings (*e.g.,* no wet rales on lung auscultation, no pharyngeal congestion, no lymphadenopathy); iii) normal laboratory test results (WBC and neutrophil percentage within age-specific reference ranges for children, CRP <10 mg/L, PCT <0.5 ng/mL); iv) absence of underlying diseases (*e.g.,* congenital heart disease, immunodeficiency, chronic lung disease) and no history of hospitalization within the past year; v) gender matching based on the age stratification of the case groups to ensure balanced age and gender distribution between the control and case groups, thereby minimizing confounding factors.

Exclusion criteria: (1) Congenital immunodeficiency diseases; (2) Use of immunosuppressive agents within 1 month before enrollment; (3) Presence of chromosomal abnormalities; (4) Incomplete clinical data; (5) In the CG, a history of infection within the past 3 months.

This single-center retrospective study enrolled subjects from the pediatric intensive care unit (PICU) of Huai’an Maternal and Child Health Hospital, affiliated with Yangzhou University. As a regional maternal and child health specialist hospital in Northern Jiangsu, this center admits over 2,000 pediatric patients with infectious diseases annually, covering various types including bacterial and viral infections. Its service scope extends to Huai’an and surrounding areas such as Yancheng and Lianyungang. The distribution of patient age and disease severity is reasonably representative, allowing the study to reflect, to a certain extent, the clinical characteristics and inflammatory marker profiles of bacterial pneumonia and sepsis in children within the Northern Jiangsu region.

Sample size estimation: Based on a pilot study showing an AUC of 0.82 for PCT in discriminating sepsis, with *α* = 0.05 and *β* = 0.2, the minimum sample size calculated using *PASS 15.0* was 85 cases per group. The sample size in this study meets the statistical requirements.

**Informed consent:** Informed consent was obtained from all individuals included in this study, or their legal guardians or wards.

**Ethical approval:** The research related to human use has been complied with all the relevant national regulations, institutional policies and in accordance with the tenets of the Helsinki Declaration, and has been approved by the Ethics Committee of the Huai’an Maternal and Child Health Care Hospital Affiliated to Yangzhou University (Approval No. 2025038).

### 2.2 Clinical observation indicators

Basic information was collected. Data on sex, age, and BMI were extracted from the hospital electronic medical record system, along with past medical history (allergy history, vaccination history, hospitalization history within the past year). Disease course was defined as the period from the onset of initial symptoms (*e.g.,* fever, cough) to the time of enrollment. Disease Severity Assessment: Disease severity was assessed in the sepsis group using the pSOFA score (range 0–12, score ≥ 2 indicated organ dysfunction, with higher scores reflecting greater severity). The bacterial pneumonia group was assessed using the dedicated severity grading criteria for childhood community-acquired pneumonia. Comorbidities included congenital heart disease, bronchial asthma, premature birth history (gestational age <37 weeks), and immunodeficiency disorders (*e.g.,* primary immunodeficiency, secondary immunodeficiency due to long-term use of corticosteroids/immunosuppressants). Antibiotic use history: Recorded whether antibiotics (e.g., penicillins, cephalosporins, macrolides) were used within 72 h prior to enrollment and the duration of use (<24 h, 24–48 h, 48–72 h).

Inflammatory marker testing was conducted. Fasting venous blood (3 mL) was collected in the morning on the day of enrollment. Of this, 2 mL was placed in an EDTA anticoagulant tube for complete blood count analysis, and 1 mL was placed in a coagulation-promoting tube for CRP and PCT testing. Samples were delivered to the laboratory within 2 h after collection to avoid hemolysis affecting the results.

Laboratory Measurements: CRP was measured using immunoturbidimetry on a Beckman Coulter AU5800 automatic biochemical analyzer. PCT was measured using electrochemiluminescence immunoassay on a Roche e601 analyzer. Peripheral blood WBC, NE#, and NE% were measured using impedance methods combined with flow cytometry on a Sysmex XN-9000 automated hematology analyzer. All tests were performed using instrument-specific reagents, strictly adhering to standard laboratory operating procedures.

Quality Control: Quality control measures included: ① Internal Quality Control: Daily calibration using instrument-specific quality control materials before testing ensured coefficients of variation (CV) < 5 % for CRP and PCT, and <3 % for hematological parameters. Sample testing proceeded only after passing quality control. ② External Quality Assessment: The laboratory participated quarterly in the external quality assessment scheme of the National Center for Clinical Laboratories. Results for CRP, PCT, and hematological parameters were all acceptable, with results traceable to international standards. ③ Abnormal Result Verification: Samples with results exceeding three times the upper reference limit were recollected and retested. If the difference between the two results was <10 %, the average was taken; if >10 %, the result was verified by a senior laboratory technician.

### 2.3 Statistical methods

Statistical analysis was performed using *SPSS 26.0*, and graphs were generated with *GraphPad Prism 9.0*. For continuous variables, the Shapiro–Wilk test was first applied to assess normality. Data conforming to a normal distribution were expressed as mean ± standard deviation (x̄ ± *s*) and were compared between two groups using the independent samples *t*-test, while comparisons among multiple groups were conducted using one-way analysis of variance (one-way ANOVA), with post-hoc pairwise comparisons performed using the LSD-t test. Non-normally distributed data were expressed as median (interquartile range) [M (Q1, Q3)], and comparisons between two groups were made using the Mann–Whitney *U* test, with the Kruskal–Wallis *H* test used for comparisons among multiple groups. Categorical data were expressed as number (percentage) [*n* (%)] and were compared using the chi-square test. When the theoretical frequency was less than 5, Fisher’s exact test was applied. Statistical Analysis: Multivariable logistic regression analysis (adjusting for age, sex, and BMI) was employed to explore the associations between plasma inflammatory injury markers (CRP, PCT, WBC, NE#) and the risk of bacterial pneumonia and sepsis in children, and to identify independent risk factors for these two conditions. Diagnostic performance was evaluated by plotting receiver operating characteristic (ROC) curves for each inflammatory marker. The area under the curve (AUC), optimal diagnostic cut-off value (determined by the maximum Youden index method, where Youden index = sensitivity + specificity - 1), sensitivity (Sen), specificity (Spe), and 95 % confidence interval (95 % CI) were calculated. The diagnostic performance of the AUC was categorized as follows: AUC > 0.9 indicated excellent diagnostic performance, 0.7 < AUC ≤ 0.9 indicated good diagnostic performance, 0.5 < AUC ≤ 0.7 indicated moderate diagnostic performance, and AUC = 0.5 indicated no diagnostic value. All statistical tests in this study were two-sided, and a *P*-value < 0.05 was considered statistically significant.

## 3 Results

### 3.1 Contrast of basic information of subjects

Comparison of baseline characteristics revealed no significant differences among the three groups in terms of age, gender distribution, BMI, allergy history, or proportion with incomplete vaccination (*P* > 0.05), while key differences were observed between the SG and the BPG (Table 1).

**Table 1:** Comparison of baseline characteristics of the study subjects.

Characteristic	SG (*n* = 116)	BPG (*n* = 126)	CG (*n* = 65)	Statistical value	*P*
Age (months), x̄ ± *s*	42.5 ± 18.7	40.2 ± 17.9	39.8 ± 16.5	*F* = 0.52	0.595
Sex (male), *n* (%)	65 (56.0)	68 (54.0)	35 (53.8)	*χ* ^2^ = 0.15	0.927
BMI (kg/m^2^), x̄ ± *s*	15.92 ± 2.89	16.35 ± 2.57	16.83 ± 2.31	*F* = 2.87	0.058
pSOFA score, M (Q1, Q3)	4.0 (2.0, 6.0)	–	–	–	–
CURB-14 score, M (Q1, Q3)	–	1.0 (0.0, 2.0)	–	–	–
Severe cases (score ≥ 2), *n* (%)	79 (68.1)	36 (28.6)	–	*χ* ^2^ = 39.45	<0.001
Comorbidities, *n* (%)	41 (35.3)	23 (18.3)	0 (0.0)	*χ* ^2^ = 19.76	<0.001
Congenital heart disease	14 (12.1)	7 (5.6)	0 (0.0)	*χ* ^2^ = 3.98	0.046
Immunodeficiency disorder	11 (9.5)	3 (2.4)	0 (0.0)	*χ* ^2^ = 6.73	0.01
Antibiotic use within 72 h before enrollment, *n* (%)	73 (62.9)	57 (45.2)	0 (0.0)	*χ* ^2^ = 18.52	<0.001
Duration > 48 h	28 (38.8)	13 (22.8)	–	*χ* ^2^ = 4.27	0.039
Prior hospitalization history, *n* (%)	56 (48.3)	38 (30.2)	2 (3.1)	*χ* ^2^ = 37.81	<0.001
Disease duration (days), x̄ ± *s*	7.81 ± 3.42	5.23 ± 2.14	–	*t* = 6.85	<0.001

Regarding disease severity scores, the median pSOFA score in the SG was 4.0 (Q1 = 2.0, Q3 = 6.0), with 68.1 % (79/116) of children having a score ≥ 4; the BPG had a median CURB-14 score of 1.0 (Q1 = 0.0, Q3 = 2.0), with 28.6 % (36/126) having a score ≥ 2, and the difference between the two groups was statistically significant (χ^2^ = 39.45, *P* < 0.001). The overall incidence of comorbidities in the SG (35.3 %, 41/116) was significantly higher than that in the BPG (18.3 %, 23/126) (χ^2^ = 8.21, *P* = 0.004), particularly for congenital heart disease (12.1 % vs. 5.6 %, χ^2^ = 3.98, *P* = 0.046) and immunodeficiency disorders (9.5 % vs. 2.4 %, χ^2^ = 6.73, *P* = 0.010). The rate of antibiotic use within 72 h prior to enrollment was 62.9 % (73/116) in the SG, significantly higher than the 45.2 % (57/126) in the BPG (χ^2^ = 7.93, *P* = 0.005), and the proportion of patients with antibiotic use duration exceeding 48 h was also higher in the SG (38.8 % vs. 22.8 %, χ^2^ = 4.27, *P* = 0.039). Additionally, the proportion of patients with prior hospitalization in the SG (48.3 %, 56/116) was significantly higher than that in the BPG (30.2 %, 38/126) and the CG (3.1 %, 2/65) (*P* < 0.001), and the disease duration in the SG (7.81 ± 3.42 days) was significantly longer than that in the BPG (5.23 ± 2.14 days) (t = 6.85, *P* < 0.001). The CG had no comorbidities, and no disease duration was recorded. All comparisons involving multiple groups or multiple indicators in this study were corrected for multiple testing using the Bonferroni method. The inter-group differences in the baseline data mentioned above remained statistically significant after correction (adjusted *P* < 0.05).

### 3.2 Contrast of plasma inflammatory factors in subjects

**Figure 1:**
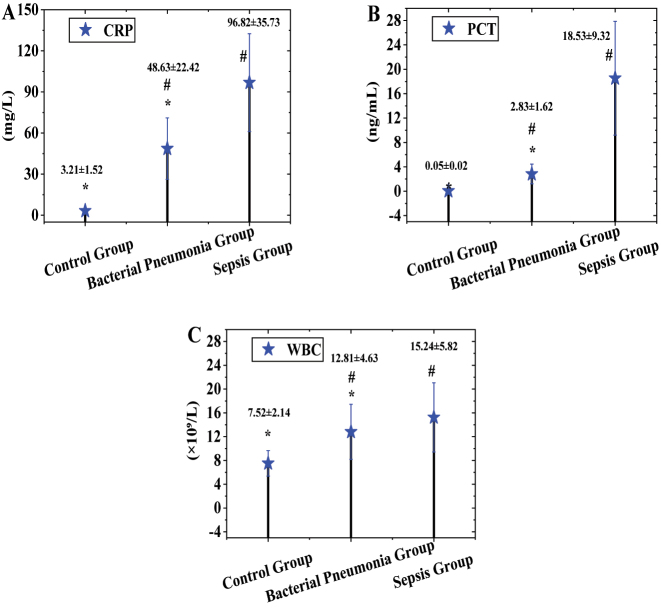
Contrast of plasma inflammatory factors (CRP, PCT, WBC) in subjects. (A-C represent CRP, PCT, and WBC, respectively). Note: * as against the SG, # as against the CG, *P* *< *0.05.

**Figure 2:**
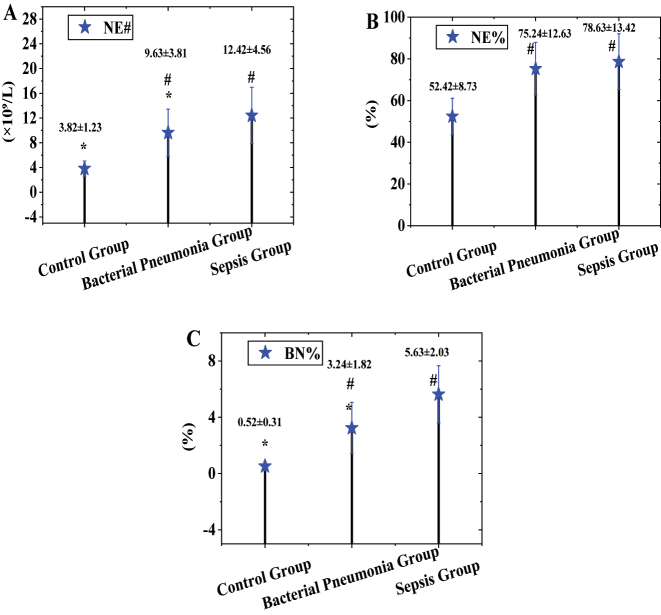
Contrast of plasma inflammatory factors (NE#, NE%, and BN%) in subjects. (A-C represent NE#, NE%, and BN%, respectively). Note: * as against the SG, # as against the CG, *P < *0.05.

Compared to the CG, the levels of inflammatory markers (CRP, PCT, WBC, NE#, and NE%) were significantly elevated in both the BPG and SG. These markers were notably higher in the SG than in the BPG (*P* < 0.05), with the most pronounced difference observed in PCT. No statistically significant difference in NE% was found between the BPG and SG (*P* > 0.05) (Figures 1 and 2). Comparisons of inflammatory factor levels among multiple groups remained statistically significant after Bonferroni correction (adjusted *P* < 0.05).

### 3.3 Correlation analysis of inflammatory factors with the occurrence of bacterial pneumonia

Multivariable Logistic Regression Analysis: Multivariable logistic regression analysis (adjusted for age, sex, BMI) showed that CRP, PCT, WBC, and NE# were all independent risk factors for the occurrence of bacterial pneumonia in children (all *P* < 0.01). Among these, PCT had the strongest predictive value (OR = 2.15, 95 % CI: 1.78–2.60, Wald = 15.67, *P* < 0.001). NE% and BN% showed no significant association with the occurrence of bacterial pneumonia in children (all *P* > 0.05), and their odds ratio 95 % confidence intervals included 1, indicating no independent predictive value (Table 2). Note: These results were corrected for multiple testing using the Bonferroni method and remained statistically significant after correction (adjusted *P* < 0.01).

**Table 2:** Correlation analysis of inflammatory factors with the occurrence of bacterial pneumonia.

Indicators	OR value (95%CI)	Wald value	Adjusted *P* values *
CRP	1.82 (1.45–2.28)	12.34	<0.001
PCT	2.15 (1.78–2.60)	15.67	<0.001
WBC	1.63 (1.32–2.01)	10.25	0.002
NE#	1.75 (1.48–2.07)	13.42	<0.001
NE%	1.12 (0.95–1.32)	1.53	0.216
BN%	1.08 (0.92–1.27)	1.21	0.271

### 3.4 Correlation analysis of inflammatory factors with the occurrence of sepsis

Multivariable logistic regression analysis (adjusted for age, sex, and BMI) revealed that CRP, PCT, WBC, and NE# were independent risk factors for sepsis (all *P* < 0.001). Among these, PCT demonstrated the strongest association and predictive value (OR = 3.42, 95 % CI: 2.76–4.24, Wald = 24.56, *P* < 0.001). In contrast, the associations of NE% and BN% with sepsis occurrence did not reach statistical significance (NE%: OR = 1.25, 95 % CI: 0.98–1.59, *P* = 0.077; BN%: OR = 1.18, 95 % CI: 0.97–1.43, *P* = 0.09). The 95 % confidence intervals for their OR values both included 1, suggesting that these indicators may not be independent predictors of sepsis (Table 3). This study used the Bonferroni method for multiple testing correction, and the above results remained statistically significant after correction (adjusted *P* < 0.01).

**Table 3:** Correlation analysis of inflammatory factors with the occurrence of sepsis.

Indicators	OR value (95%CI)	Wald value	Adjusted *P* values *
CRP	2.35 (1.92–2.88)	18.23	<0.001
PCT	3.42 (2.76–4.24)	24.56	<0.001
WBC	2.04 (1.68–2.48)	16.34	<0.001
NE#	2.28 (1.87–2.78)	19.45	<0.001
NE%	1.25 (0.98–1.59)	3.12	0.077
BN%	1.18 (0.97–1.43)	2.87	0.09

### 3.5 Diagnostic efficacy of inflammatory injury factors for sepsis

**Figure 3:**
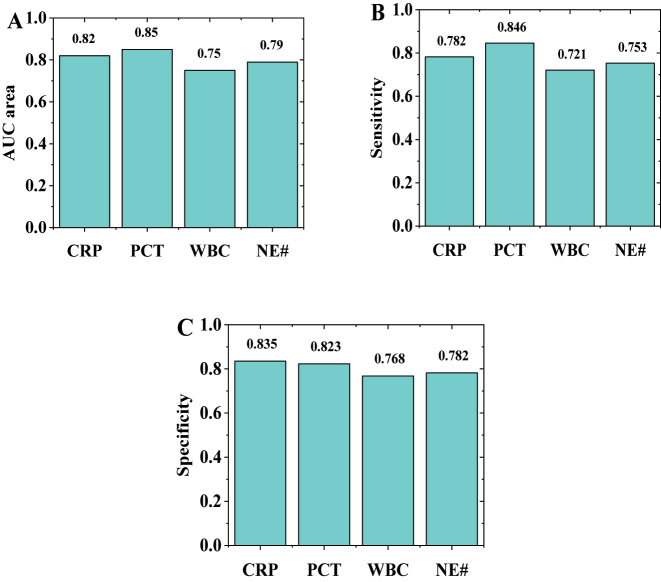
Diagnostic performance of inflammatory injury markers for sepsis. (A: AUC; B: sensitivity; C: specificity).

The diagnostic efficacy analysis showed that all inflammatory indicators had good diagnostic value for sepsis. PCT had the best diagnostic efficacy, with an AUC of 0.85 (95%CI: 0.81–0.89). When the cutoff value was 5.8 ng/mL, its Sen and Spe were 84.6 % and 82.3 %. CRP had an AUC of 0.82 (95%CI: 0.78–0.86). With a cutoff value of 28.5 mg/L, its Sen and Spe were 78.2 % and 83.5 %. The AUC for WBC and NE# were 0.75 (95%CI: 0.70–0.80) and 0.79 (95%CI: 0.74–0.84). The corresponding Sen were 72.1 % and 75.3 %, and the Spe were 76.8 % and 78.2 % (Table 4 and Figure 3).

**Table 4:** ROC curve parameters of inflammatory markers for diagnosing sepsis.

Indicator	AUC (95%CI)	Optimal cut-off	Sen (95%CI)	Spe (95%CI)	+LR (95%CI)	-LR (95%CI)	PPV (95%CI)	NPV (95%CI)
PCT	0.85 (0.81–0.89)	5.8 ng/mL	84.6 % (77.2%–90.1 %)	82.3 % (75.5%–87.6 %)	4.78 (3.52–6.51)	0.19 (0.12–0.29)	79.5 % (71.8%–85.6 %)	86.7 % (80.2%–91.5 %)
CRP	0.82 (0.78–0.86)	28.5 mg/L	78.2 % (70.3%–84.7 %)	83.5 % (76.8%–88.7 %)	4.75 (3.48–6.48)	0.26 (0.18–0.37)	80.1 % (72.2%–86.3 %)	81.5 % (74.6%–86.9 %)
WBC	0.75 (0.70–0.80)	15.2 × 10^9^/L	72.1 % (63.8%–79.2 %)	76.8 % (69.5%–82.9 %)	3.11 (2.35–4.12)	0.36 (0.28–0.47)	70.3 % (61.9%–77.6 %)	78.3 % (70.9%–84.4 %)
NE#	0.79 (0.74–0.84)	10.8 × 10^9^/L	75.3 % (67.2%–82.1 %)	78.2 % (71.0%–84.2 %)	3.45 (2.61–4.56)	0.32 (0.24–0.42)	72.8 % (64.5%–79.8 %)	80.3 % (73.0%–85.9 %)

DeLong’s test results showed that the AUC of PCT was significantly higher than that of WBC (*P* < 0.001) and NE# (*P* = 0.003), while no statistically significant difference was observed compared to CRP (*P* = 0.176). The AUC of CRP was significantly higher than that of WBC (*P* = 0.008), but not significantly different from NE# (*P* = 0.214). There was no statistically significant difference in AUC between WBC and NE# (*P* = 0.352).

Optimal diagnostic cut-off determination and validation: Using the maximum Youden index method, the Youden index reached its peak value of 0.669 at a PCT concentration of 5.8 ng/mL (Sen = 84.6 % + Spe = 82.3 % - 1 = 0.669), indicating the best balance between sensitivity and specificity at this threshold. Internal validation via the Bootstrap method (1,000 resamples) yielded a validated sensitivity of 83.2 % (95 % CI: 75.8%–88.9 %) and specificity of 81.5 % (95 % CI: 74.7–86.8 %). The difference from the original results was less than 2 %, suggesting good stability of this cut-off value for clinical differentiation of sepsis. Diagnostic efficacy: Comparisons of the diagnostic efficacy of each indicator and the results of the DeLong test remained statistically significant after Bonferroni correction for multiple testing (adjusted *P* < 0.05). This applied to both relevant comparisons in the study.

### 3.6 Diagnostic efficacy of inflammatory injury factors for bacterial pneumonia

The results of the diagnostic efficacy analysis for bacterial pneumonia showed that CRP had the best diagnostic efficacy, with an AUC of 0.76 (95%CI: 0.71–0.81). When 20.0 mg/L was used as the diagnostic cutoff value, its Sen and Spe were 72.5 % and 79.3 %. The AUC for NE# was 0.74 (95%CI: 0.69–0.79). With a cutoff value of 6.5 × 10^9^/L, its Sen and Spe were 73.1 % and 76.2 %. The AUC for WBC and PCT were relatively lower, at 0.72 (95%CI: 0.67–0.77) and 0.70 (95%CI: 0.65–0.75). The corresponding Sen were 70.2 % and 68.4 %, and the Spe were 73.8 % and 75.6 % (Table 5).

**Table 5:** ROC curve parameters of inflammatory markers for diagnosing bacterial pneumonia.

Indicator	AUC (95%CI)	Optimal cut-off	Sen (95%CI)	Spe (95%CI)	+LR (95%CI)	-LR (95%CI)	PPV (95%CI)	NPV (95%CI)
CRP	0.76 (0.71–0.81)	20.0 mg/L	72.5 % (64.1%–79.8 %)	79.3 % (72.0%–85.3 %)	3.51 (2.63–4.68)	0.35 (0.27–0.45)	76.8 % (68.3%–83.8 %)	75.1 % (67.5%–81.5 %)
NE#	0.74 (0.69–0.79)	6.5 × 10^9^/L	73.1 % (64.7%–80.3 %)	76.2 % (68.7%–82.5 %)	3.07 (2.31–4.08)	0.35 (0.27–0.46)	73.5 % (65.0%–80.7 %)	75.8 % (68.2%–82.3 %)
WBC	0.72 (0.67–0.77)	12.8 × 10^9^/L	70.2 % (61.6%–77.7 %)	73.8 % (66.1%–80.4 %)	2.68 (2.03–3.55)	0.40 (0.32–0.51)	70.5 % (61.8%–78.2 %)	73.5 % (65.7%–80.2 %)
PCT	0.70 (0.65–0.75)	1.5 ng/mL	68.4 % (59.6%–76.2 %)	75.6 % (68.0%–82.1 %)	2.80 (2.10–3.75)	0.42 (0.34–0.53)	71.2 % (62.3%–78.9 %)	72.7 % (65.0%–79.4 %)

DeLong’s test results showed that the AUC of CRP was significantly higher than that of PCT (*P* = 0.004), while no statistically significant differences were observed compared to WBC (*P* = 0.138) or NE# (*P* = 0.365). The AUC of NE# was significantly higher than that of PCT (*P* = 0.012), but not significantly different from WBC (*P* = 0.287). No statistically significant difference in AUC was found between WBC and PCT (*P* = 0.415).

**Figure 4:**
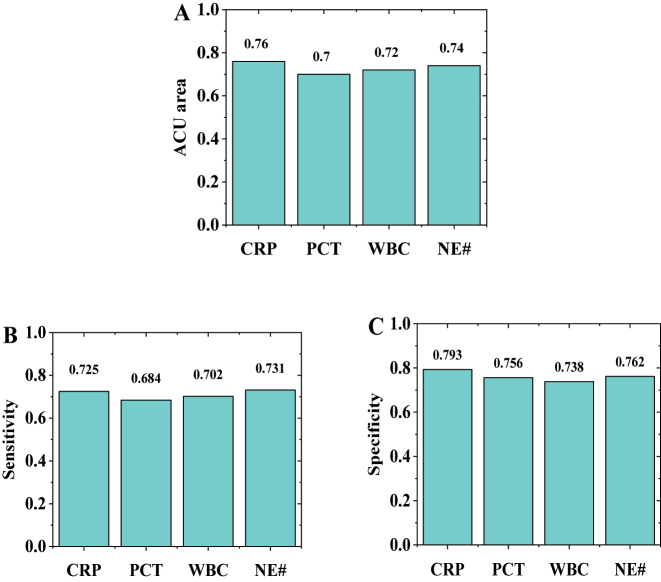
Diagnostic performance of inflammatory injury markers for bacterial pneumonia. (A: AUC; B: sensitivity; C: specificity).

Optimal diagnostic cut-off determination and validation: Using the maximum Youden index method, the Youden index reached its peak value of 0.518 at a CRP concentration of 20.0 mg/L (Sen = 72.5 % + Spe = 79.3 % - 1 = 0.518), indicating a balanced ability to identify and rule out bacterial pneumonia at this threshold. Internal validation via the Bootstrap method (1,000 resamples) yielded a validated sensitivity of 71.3 % (95 % CI: 62.8%–78.7 %) and specificity of 78.6 % (95 % CI: 71.2%–84.5 %). The difference from the original results was less than 2 %, confirming reliable threshold stability (Figure 4). After comparison of the diagnostic efficacy of each index and the DeLong test, the difference remained statistically significant after Bonferroni correction for multiple testing (adjusted *P* < 0.05).

## 4 Discussion

Bacterial pneumonia and sepsis are two critical infectious diseases in pediatric clinical practice, with highly overlapping clinical manifestations posing a significant challenge for early differential diagnosis [11], [12], [13]. This study provided experimental evidence for the precise differentiation of these two conditions by analyzing the clinical characteristics and inflammatory marker expression profiles in affected children. From a pathophysiological perspective, the differences in inflammatory marker expression between the two diseases fundamentally reflect the pathological distinction between local infection and systemic inflammatory response: bacterial pneumonia is centered on local pulmonary inflammation, with pathological processes primarily involving neutrophil infiltration and local pro-inflammatory factor release, resulting in moderately elevated inflammatory markers [14], 15]. In contrast, sepsis involves systemic dissemination of inflammatory mediators, leading to endothelial damage, microcirculatory disturbances, and multi-organ dysfunction, manifested by significantly elevated markers like procalcitonin (PCT), which underscores the clinical value of PCT as a marker of systemic infection [16]. This study found that the absolute neutrophil count (NE#) was significantly elevated in both diseases, whereas the neutrophil percentage (NE%) showed no significant inter-group difference. Furthermore, NE# demonstrated superior diagnostic efficacy compared to NE% and was confirmed as an independent risk factor for disease occurrence, suggesting that NE# reflects the intensity of the inflammatory response in pediatric bacterial infections more objectively than the percentage indicator [17]. Existing research on the diagnostic value of neutrophil-related indicators remains controversial, often focusing only on NE% changes without simultaneously analyzing NE#. By directly comparing their ROC curve parameters, this study further confirmed the advantage of NE# in assessing pediatric bacterial infections.

The baseline characteristics analyzed in this study also provided clues for the clinical differentiation and risk stratification of the two diseases. The sepsis group had significantly higher disease severity scores, comorbidity rates, rates and duration of pre-enrollment antibiotic use, a higher proportion of previous hospitalization history, and a longer disease course compared to the bacterial pneumonia group. These clinical features might be associated with the progression of infection towards systemic inflammation, suggesting that enhanced monitoring is needed for children with such characteristics. However, it is important to note that the analysis of baseline characteristics was descriptive and did not fully adjust for confounding factors like socioeconomic status or severity of underlying diseases. The independent association of these characteristics with disease occurrence and progression requires validation in subsequent studies. Additionally, the slightly lower mean BMI in the sepsis group compared to controls suggested a potential link between nutritional status and infection severity, although the causal relationship remains unclear and warrants further investigation [18], 19]. By combining the Youden index maximum method with Bootstrap internal validation, this study established differentiated diagnostic thresholds for PCT and C-reactive protein (CRP) to distinguish between bacterial pneumonia and sepsis. These thresholds can serve as references for differentiating local from systemic bacterial infections in clinical practice. When a child’s PCT and CRP levels fall within the critical range, a comprehensive judgment integrating clinical symptoms, signs, and other laboratory indicators is necessary. Compared with other studies, the diagnostic threshold for PCT in sepsis identified in this study differed from that reported by Moisa et al. [20] in 2023, who proposed a PCT cut-off of 1.49 ng/mL for bacterial sepsis, lower than the 5.8 ng/mL found here. This discrepancy likely stems from differences in study populations and infection types: Moisa et al. focused on differentiating bacterial from viral sepsis, whereas this study compared local versus systemic bacterial infections in pediatrics. The differing pathophysiological features led to different marker thresholds. Furthermore, Zhang et al. [21] in 2023, in their study on bacterial pneumonia and sepsis after cardiovascular surgery, did not use PCT as a core predictor but instead constructed the DICS-I model including IL-6 and mechanical ventilation duration. This suggests that the diagnostic value of inflammatory markers is context-specific; in special situations like post-operative infection, underlying diseases and therapeutic interventions can influence marker expression, making a single marker insufficient for diagnostic needs.

Regarding clinical course and prognosis-related features, the disease duration was significantly longer in the sepsis group than in the bacterial pneumonia group, possibly reflecting the more complex pathophysiology of sepsis and longer recovery period for children. The finding that the proportion of children with a history of previous hospitalization was significantly higher in the sepsis group aligns with the conclusions of Kang et al. [22] in 2022, suggesting that children with recurrent infections are more prone to the systemic progression of infection to sepsis. Enhanced infection surveillance is clinically necessary for such high-risk children. This study also found a lower mean BMI in the sepsis group compared to controls, hinting at a potential association between malnutrition and infection severity. However, as age, dietary habits, and other confounding factors were not adjusted for, a causal relationship could not be confirmed. From a clinical application perspective, the single-center results of this study offer preliminary insights for stratified diagnosis of pediatric bacterial infections [23], 24]: For children with suspected infection, CRP and white blood cell count could be used for initial screening. For those with high suspicion of sepsis, adding PCT measurement could enhance diagnostic accuracy. For high-risk children with prolonged disease duration, malnutrition, or a history of recurrent hospitalization, dynamic monitoring of PCT changes could aid in the early identification of infection progression risk. The finding that NE# had superior diagnostic efficacy compared to NE% provides a reference for interpreting clinical laboratory indicators, suggesting clinicians should pay more attention to NE# trends. However, as this was a single-center exploratory study, the clinical superiority of NE# requires further validation through large-scale, multi-center studies.

This study has several limitations, and its conclusions should be interpreted with caution. First, as a single-center retrospective study with a limited sample size, it is subject to selection bias and did not fully incorporate confounding factors like socioeconomic status or disease severity scores, limiting the external validity of the findings. Second, the diagnostic thresholds were validated only through internal Bootstrap, lacking prospective, multi-center external validation, posing a risk of overfitting. Third, only single time-point biomarker data at enrollment were collected, preventing analysis of their dynamic changes in relation to disease severity or treatment response. Fourth, pathogen typing analysis was not performed, and outcome indicators like antibiotic efficacy or patient prognosis were not collected, making it difficult to explore the markers’ value in guiding treatment or assessing prognosis. Based on these limitations, the clinical diagnostic suggestions are exploratory and cannot be directly applied as clinical standards. Future research should address these points: first, conduct large-scale, multi-center prospective studies to validate the diagnostic thresholds proposed here, enhancing clinical applicability. Second, incorporate dynamic biomarker monitoring data to analyze associations with disease progression and treatment response, clarifying their value in efficacy assessment and prognosis. Third, combine pathogen typing to explore inflammatory marker expression profiles in infections caused by different bacteria, providing a basis for precision anti-infective therapy. Fourth, develop combined prediction models integrating clinical features and inflammatory markers to improve differential diagnostic efficacy for pediatric bacterial pneumonia and sepsis. Fifth, conduct interventional studies to evaluate the actual impact of diagnostic strategies based on these markers on clinical decision-making, rational antibiotic use, and patient outcomes. In summary, based on single-center retrospective data, this study preliminarily explored the differential diagnostic value of PCT, CRP, and NE# for pediatric bacterial pneumonia and sepsis. It identified the diagnostic advantage of NE# over NE% and established differentiated diagnostic thresholds for the two conditions, providing a reference for early clinical differentiation. By comparing with existing studies, it further clarified the context-specific nature of inflammatory markers’ diagnostic value, offering directions for future research. However, limited by the study design and sample size, these findings remain exploratory. The clinical value of the markers and the applicability of the thresholds require validation through multi-center prospective studies. In clinical practice, a comprehensive assessment integrating the child’s clinical features, laboratory indicators, and imaging findings is essential, avoiding over-reliance on a single biomarker or diagnostic threshold to improve diagnostic accuracy for pediatric bacterial infections and optimize treatment decisions.

## 5 Conclusions

By analyzing the expression characteristics of plasma inflammatory injury markers and clinical data in children with bacterial pneumonia and sepsis, this study clarified the differences in PCT, CRP, and NE# levels between the two conditions. It confirmed that PCT possesses good differential diagnostic value for sepsis, that differentiated CRP cut-off values can reflect the extent and severity of bacterial infection to some degree, and that NE# reflects the intensity of the inflammatory response in pediatric bacterial infections more objectively than NE%. These findings provide a reference for interpreting relevant clinical indicators. Concurrently, the study identified clinical features more prevalent in the sepsis group, such as longer disease duration, higher rates of prior hospitalization, and higher comorbidity rates, offering objective evidence for clinical differentiation and identification of high-risk populations in pediatric bacterial infections. The preliminary findings provide an experimental reference for the early differential diagnosis of pediatric bacterial pneumonia and sepsis and offer insights for optimizing the selection of inflammatory assessment indicators in clinical practice: primary healthcare facilities could use CRP and WBC counts as basic screening tools for pediatric bacterial infections. For children with suspected sepsis in higher-level medical institutions, adding PCT measurement might improve diagnostic accuracy. Concurrently, enhanced infection surveillance is needed for children with high-risk features like recurrent infection history or malnutrition.

However, as this was a single-center retrospective exploratory study with a limited sample size, and the diagnostic thresholds were only internally validated without external prospective validation, the clinical applicability of the conclusions requires further confirmation through large-scale, multi-center prospective studies. Future high-quality research is needed to validate the diagnostic value and threshold applicability of these inflammatory markers. Constructing combined prediction models integrating pathogen typing and dynamic marker monitoring is essential to provide more reliable evidence for the precise diagnosis and treatment of pediatric bacterial infections. In clinical practice, a comprehensive evaluation combining the child’s clinical symptoms, signs, imaging findings, and laboratory indicators remains necessary, avoiding over-reliance on any single biomarker.
